# Runx1 is upregulated by STAT3 and promotes proliferation of neonatal rat cardiomyocytes

**DOI:** 10.14814/phy2.15872

**Published:** 2023-12-01

**Authors:** Shota Suzuki, Shota Tanaka, Yusuke Kametani, Ayaka Umeda, Kosuke Nishinaka, Kaho Egawa, Yoshiaki Okada, Masanori Obana, Yasushi Fujio

**Affiliations:** ^1^ Laboratory of Clinical Science and Biomedicine, Graduate School of Pharmaceutical Sciences Osaka University Suita City Osaka Japan; ^2^ Center for Infectious Disease Education and Research (CiDER) Osaka University Suita City Osaka Japan; ^3^ Integrated Frontier Research for Medical Science Division, Institute for Open and Transdisciplinary Research Initiative (OTRI) Osaka University Suita City Osaka Japan; ^4^ Global Center for Medical Engineering and Informatics (MEI) Osaka University Suita City Osaka Japan; ^5^ Radioisotope Research Center, Institute for Radiation Sciences Osaka University Suita City Osaka Japan

**Keywords:** cardiomyocytes, cell dedifferentiation, cell proliferation, RNA‐seq, Runx1

## Abstract

Though it is well known that mammalian cardiomyocytes exit cell cycle soon after birth, the mechanisms that regulate proliferation remain to be fully elucidated. Recent studies reported that cardiomyocytes undergo dedifferentiation before proliferation, indicating the importance of dedifferentiation in cardiomyocyte proliferation. Since Runx1 is expressed in dedifferentiated cardiomyocytes, Runx1 is widely used as a dedifferentiation marker of cardiomyocytes; however, little is known about the role of Runx1 in the proliferation of cardiomyocytes. The purpose of this study was to clarify the functional significance of Runx1 in cardiomyocyte proliferation. qRT‐PCR analysis and immunoblot analysis demonstrated that Runx1 expression was upregulated in neonatal rat cardiomyocytes when cultured in the presence of FBS. Similarly, STAT3 was activated in the presence of FBS. Interestingly, knockdown of STAT3 significantly decreased Runx1 expression, indicating Runx1 is regulated by STAT3. We next investigated the effect of Runx1 on proliferation. Immunofluorescence microscopic analysis using an anti‐Ki‐67 antibody revealed that knockdown of Runx1 decreased the ratio of proliferating cardiomyocytes. Conversely, Runx1 overexpression using adenovirus vector induced cardiomyocyte proliferation in the absence of FBS. Finally, RNA‐sequencing analysis revealed that Runx1 overexpression induced upregulation of cardiac fetal genes and downregulation of genes associated with fatty acid oxidation. Collectively, Runx1 is regulated by STAT3 and induces cardiomyocyte proliferation by juvenilizing cardiomyocytes.

## 1 INTRODUCTION

Cardiovascular disease is the leading cause of death in the world. Since mammalian cardiomyocytes are terminally differentiated cells that have largely lost their proliferative capacity (Porrello et al., 2011), the heart exhibits the limited regenerative capacity. So, once cardiomyocytes are lost, cardiac function declines irreversibly leading to heart failure. Therefore, elucidating the mechanisms that regulate cardiomyocyte proliferation may provide novel insights into the development of therapeutic strategies of heart failure (Bergmann et al., 2015).

While adult mammalian cardiomyocytes have little proliferative potential, proliferative capacity is maintained in adult zebrafish. Therefore, a number of studies have been performed to address the molecular mechanisms of cardiomyocyte proliferation using zebrafish model. In zebrafish, cardiomyocyte proliferation is induced in response to heart dissection, and the dissected heart is eventually repaired. (Poss et al., 2002). In this process, it has been reported that cardiomyocyte dedifferentiation occurs before proliferation (Jopling et al., 2010). Dedifferentiation is characterized by the disassembly of sarcomere structures and the expression of cardiac progenitor cell marker genes (Zhu et al., 2021). In mammals, adult murine cardiomyocytes that were cocultured with neonatal rat cardiomyocytes (NRCMs) proliferated after dedifferentiation (Wang et al., 2017). Thus, much attention is being paid to the importance of dedifferentiation in proliferation.

RUNX family transcription factor 1 (Runx1) is used as a marker of mammalian cardiomyocyte dedifferentiation in recent studies (Beisaw et al., 2020; Ikeda et al., 2019; Kubin et al., 2011). While Runx1 is upregulated in dedifferentiated cardiomyocytes, the biological significance of Runx1 in cardiomyocyte proliferation is unclear. Oncostatin M (OSM), a member of interleukin‐6 (IL‐6) family cytokines, has been reported to induce dedifferentiation of mammalian cardiomyocytes (Kubin et al., 2011). OSM binds to OSM receptor or leukemia inhibitory factor receptor and activates signal transducer and activator of transcription 3 (STAT3) through glycoprotein 130 (Hermanns, 2015). Previously, we reported that STAT3 plays an important role in the healing process of myocarditis with cardiomyocyte proliferation (Miyawaki et al., 2017). However, the role of STAT3 in Runx1 expression is still unknown.

In this study, we addressed the mechanism of Runx1 expression and its significance in cardiomyocyte proliferation. Runx1 expression was upregulated in NRCM cultured in the presence of fetal bovine serum (FBS). STAT3 was activated by FBS, and knockdown of STAT3 decreased proliferating NRCMs in number. In addition, Runx1 expression was significantly decreased by STAT3 knockdown. Importantly, Runx1 knockdown significantly decreased the ratio of proliferating NRCMs, while Runx1 overexpression increased the ratio of proliferating NRCMs. Finally, RNA‐sequencing analysis revealed that the overexpression of Runx1 in cardiomyocytes induced the expression of fetal genes and suppressed that of the genes characteristic of mature cardiomyocytes. These data suggest that the induction of Runx1 may be a key event of cardiomyocyte dedifferentiation, providing a novel insight into the molecular mechanism of cardiomyocyte proliferation.

## 2 MATERIALS AND METHODS

### 2.1 NRCM culture

Animal experiments conformed to the Guide for the Care and Use of Laboratory Animals Eighth Edition updated by the US National Research Council Committee with the approval of the Animal Experimentation Committee of Osaka University and Institutional Animal Care. NRCMs were prepared from the ventricles of 1‐ to 2‐day‐old Wistar rats of both sexes, as described previously (Wahyuni et al., 2021). Specifically, hearts were digested by 0.1% collagenase type IV (Sigma‐Aldrich, C5138) and 0.1% trypsin (Gibco, 27250018). After filtrated through a cell strainer (Falcon, 352360), cells were pre‐cultured to remove cardiac fibroblasts. After 90 min, unattached cells were used as NRCMs. NRCMs are cultured in DMEM (Sigma‐Aldrich, D5796) containing 10% FBS (Gibco, A5256701) for 24 h. Before FBS stimulation, cells were washed with serum‐free medium and then cultured with DMEM containing 1% FBS for indicated time. For assays using siRNA, cells were cultured with serum‐free or 1% FBS medium for 48 h before analyses. Cells were cultured with serum‐free medium for 48 h for assays using adenovirus vector.

### 2.2 Quantitative RT‐PCR

Quantitative RT‐PCR was performed as previous described (Tomimatsu et al., 2022). Total RNA was prepared from cultured NRCM using QIAzol Lysis Reagent (QIAGEN, 79306) and subject to synthesize cDNA from 1 μg total RNA with Oligo dT (Thermo, 18418020) and ReverTra Ace (Toyobo, TRT‐101). The mRNA expression was quantified by real‐time RT‐PCR analysis (StepOne Real‐time PCR systems, Applied Biosystems) using FAST SYBR Green Master Mix (Applied Biosystems, 4385612). Primers are shown below.

*Runx1* forward, 5'‐AGTCAGATGCAGGATGCCAG‐3′; reverse, 5'‐CGAAGGCTGTCAGGTCCG‐3'.

*Gapdh* forward, 5'‐CATCACCATCTTCCAGGAGCG‐3′, reverse, 5'‐GAGGGGCCATCCACAGTCTTC‐3'.

*B2m* forward, 5'‐TGACCGTGATCTTTCTGGTGC‐3′; reverse, 5'‐AAGTTGGGCTTCCCATTCTCC‐3'.

*Acta1* forward, 5'‐CTCTTGTGTGTGACAACGGC‐3′; reverse, 5'‐ACCCATACCGACCATGACAC‐3'.

*Cnn1* forward, 5'‐ACTTTAACCGAGGTCCTGCC‐3′; reverse, 5'‐GTCGAGCTTGTTGATAAATTCGC‐3'.

*Nppa* forward, 5'‐ATCCCGTATACAGTGCGGTG‐3′; reverse, 5'‐TCAGAGAGGGAGCTAAGTGC‐3'.

*Nppb* forward, 5'‐ACAATCCACGATGCAGAAGC‐3′; reverse, 5'‐CGATCCGGTCTATCTTCTGC‐3'.

*Hand1* forward, 5'‐ACCAGCTACATCGCCTACTTG‐3′; reverse, 5'‐CAGCCAGTGCGTCCTTTAATC‐3'.

*Atp1a3* forward, 5'‐AGTGCAGGCATCAAGGTCATC‐3′; reverse, 5'‐TGAGGTCGGTGCCATGAATC‐3'.

*Ccnd1* forward, 5'‐GAGCCATGCTTAAGACTGAGGAG‐3′; reverse, 5'‐TTAGAGGCCACGAACATGCAG‐3'.

*Ccnd2* forward, 5'‐GAGAAGCTGTCCCTGATCCG‐3′; reverse, 5'‐CACTTCCTCGTCCTGCTGAAG‐3′.

### 2.3 Immunoblot analyses

Immunoblot analyses were performed as previously described (Kametani et al., 2022). Proteins were separated by SDS‐PAGE and transferred to polyvinylidene difluoride membranes (Merck Millipore, IPVH00010). The membrane was blocked for 1 h using 5% bovine serum albumin then incubated with primary antibodies overnight at 4°C. After reaction with the primary antibody, membranes were incubated with HRP‐conjugated secondary antibodies for 1 h at room temperature. Proteins were detected by ImageQuant LAS 4010 (Cytiva) using ECL Western Blotting Substrate (Promega, W1001) and Chemi‐Lumi One Super (Nacalai Tesque, 02230‐30).

For quantitative analysis of the protein expression, band intensities were measured using ImageJ software (National Institute of Health). To perform statistical analysis on the expression levels of the proteins, the results from different membranes were bridged by loading one sample into another gel.

Antibodies used in this study are shown in Table S1.

### 2.4 siRNA transfection

Twenty‐four hours after plating, NRCM were transfected with siRNA for 24 h using Lipofectamine RNAiMAX Transfection Reagent (Invitrogen, 13778‐150) according to the manufacturer's protocol. The siRNA sequence used in this study is shown in Table S2.

### 2.5 Immunofluorescence microscopic analysis

Immunofluorescence microscopic analyses were performed as previously described (Kametani et al., 2022). The cells were washed with PBS and fixed for 15 min using 4% paraformaldehyde in PBS. After permeabilization with 0.1% triton X‐100 in PBS, primary antibodies were reacted overnight at 4°C. Alexa Fluor 488‐ or Alexa Fluor 546‐conjugated secondary antibodies were reacted for 1 h in room temperature. DAPI was used for staining nuclei. Cell images were digitized by a fluorescence microscope (CV8000, Yokokawa). Images were taken from nine different fields of the single well. Total cardiomyocyte and Ki‐67^+^ or pHH3^+^ cardiomyocytes were counted by a researcher who was blinded to the assay conditions. In quantitative data, each dot presents the average ratio of nine images from the single well.

Antibodies used in this study are shown in Table S3.

### 2.6 Adenovirus vector

Adenovirus vector was prepared as previously described (Shirakura et al., 2019). The DNA fragments encoding Runx1 were inserted into the adenoviral shuttle vector pHMEF5. The shuttle vector was digested by restriction enzymes, and the expression cassette was purified and inserted to the parental adenoviral vector pAdHM4. The plasmid was linearized and transfected into HEK 293 cells using Lipofectamine 2000 (Invitrogen, 11668030). Adenoviral vectors were purified by centrifugation on a CsCl_2_ gradient. Virus titer was measured using Adeno‐X‐Rapid Titer Kit (Clontech, 632250) according to the manufacturer's protocol. Adenovirus vector expressing β‐galactosidase (β‐gal) was used as a control. Each adenovirus vector was used at 100 MOI for immunofluorescence microscopic analysis and RNA‐sequencing analysis.

### 2.7 RNA‐sequencing analysis

Total RNA was prepared from primary cultured NRCM infected with adenovirus vector expressing β‐gal or Runx1 using QIAzol and miRNeasy Micro Kit (QIAGEN, 210784). Library was prepared using a TruSeq Stranded mRNA sample prep kit (Illumina, 20020594). Sequencing was performed on an Illumina Novaseq 6000 platform in a 100‐base single‐end mode. The sequenced reads were mapped to the rat reference genome sequences (rn6) using TopHat ver. 2.1.1. in combination with Bowtie2 ver. 2.3.5.1 and SAMtools ver. 1.2. The fragments per kilobase of exon per million mapped fragments (FPKMs) were calculated using Cufflinks ver. 2.2.1. The data have been deposited with links to BioProject accession number PRJDB16862 in the DDBJ BioProject database.

### 2.8 Statistical analysis

All data are shown as mean ± SD. Comparisons between 2 groups were performed by Student's *t*‐test or Welch's *t*‐test. For multiple comparisons, one‐way ANOVA followed by Dunnett test or Tukey–Kramer test was conducted. *p* < 0.05 was considered to be statistically significant.

## 3 RESULTS

### 3.1 Serum stimulation upregulated the expression of Runx1 in NRCMs

**FIGURE 1:**
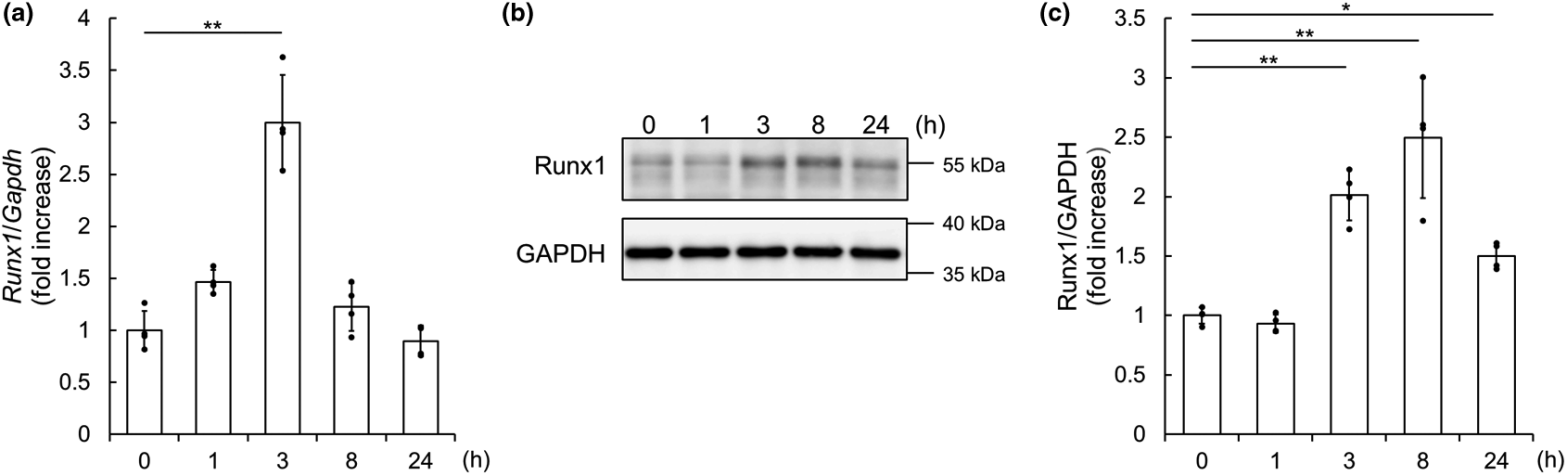
Serum stimulation upregulated Runx1 expression in neonatal rat cardiomyocytes (NRCMs). (a–c) NRCMs were stimulated by fetal bovine serum for the indicated time. (a) The expression of *Runx1* mRNA was quantified by qRT‐PCR and normalized to that of *Gapdh*. Data are shown as mean ± SD of fold change. **p < 0.01 (*n* = 4) versus 0 h by one‐way ANOVA followed by Dunnett test. (b, c) Immunoblot analysis was performed with anti‐Runx1 and anti‐GAPDH antibodies. Representative images (b) and quantification of Runx1 (c) are shown. The expression of Runx1 protein is shown as fold increase of that of control group (0 h). Data are shown as mean ± SD. *p < 0.05, **p < 0.01 (*n* = 4) versus 0 h by one‐way ANOVA followed by Dunnett test.

First, we addressed whether the expression of Runx1 is upregulated in NRCMs under proliferative condition. Since immunofluorescence microscopic analysis showed that FBS promoted the proliferation of NRCM (Figure S1A,B), as described previously (Engel et al., 2006), we examined whether the expression of Runx1 is upregulated in NRCMs by FBS. As a result, qRT‐PCR analysis revealed that the expression of *Runx1* mRNA was increased by FBS stimulation (Figure 1a). Consistently, Runx1 protein was increased in response to serum stimulation (Figure 1b,c). Thus, Runx1 expression is upregulated under the conditions that NRCMs proliferate, indicating that Runx1 is associated with NRCM proliferation.

### 3.2 Activation of STAT3 is essential for the induction of Runx1 by FBS in NRCMs

**FIGURE 2:**
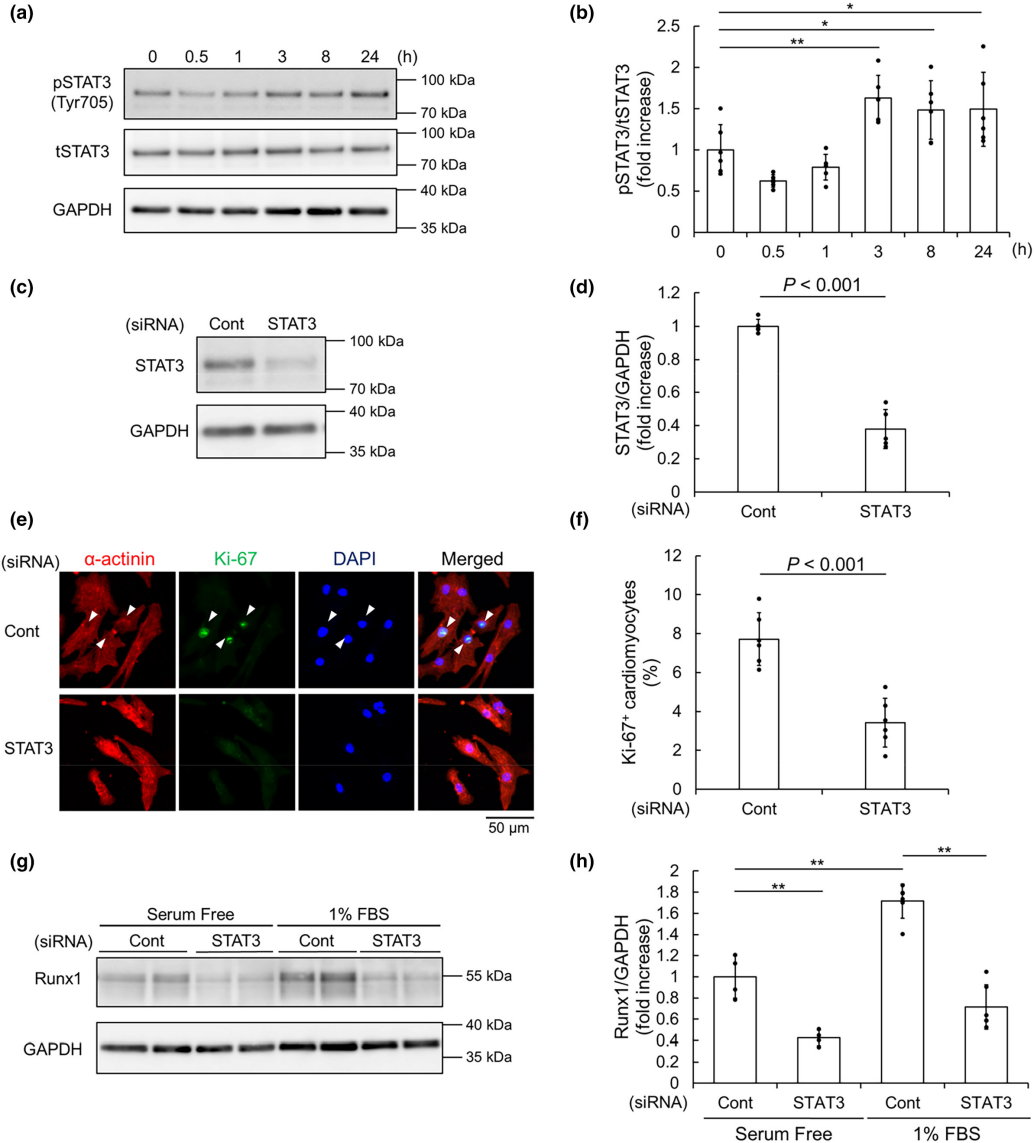
Signal transducer and activator of transcription 3 (STAT3) activation is essential for the induction of Runx1 in response to fetal bovine serum (FBS). (a, b) Neonatal rat cardiomyocytes (NRCMs) were stimulated by FBS for the indicated time. Immunoblot analysis was performed with anti‐phosphorylated STAT3 (pSTAT3), anti‐total STAT3 (tSTAT3), and anti‐GAPDH antibodies. Representative images (a) and quantification of pSTAT3 (b) are shown. Data are shown as mean ± SD of fold increase. *p < 0.05, **p < 0.01 (*n* = 6) versus 0 h by one‐way ANOVA followed by Dunnett test. (c–h) NRCMs were transfected with control siRNA (Cont) or siRNA for STAT3 at 10 nM. (c, d) Immunoblot analysis was performed with anti‐STAT3 and anti‐GAPDH antibodies. Representative images (c) and quantification of STAT3 (d) are shown. Data are shown as mean ± SD of fold increase. *p*‐value was calculated by unpaired, two‐tailed Student's *t*‐test (*n* = 5). (e, f) NRCMs were stained with anti‐sarcomeric α‐actinin and anti‐Ki‐67 antibodies. Nuclei were stained with DAPI. (e) Representative images are shown. Arrowheads indicate Ki‐67^+^ α‐actinin^+^ cells. (f) The percentage of Ki‐67^+^ α‐actinin^+^ cells per α‐actinin^+^ cells are shown. Data are shown as mean ± SD. *p*‐value was calculated by unpaired, two‐tailed Student's *t*‐test (*n* = 6). (g, h) Immunoblot analysis was performed with anti‐Runx1 and anti‐GAPDH antibodies. Representative images (g) and quantification of Runx1 (h) are shown. Data are shown as mean ± SD of fold increase. **p < 0.01 (*n* = 4 for serum‐free, *n* = 6 for 1% FBS) by one‐way ANOVA followed by Turkey‐Kramer test.

Previously, it was reported that OSM induced dedifferentiation of cardiomyocytes both in vitro and in vivo (Kubin et al., 2011). OSM is a member of IL‐6 family cytokines that transduce signals through STAT3. Thus, we hypothesized that STAT3 mediates the increase in Runx1. First, we evaluated STAT3 activities in NRCMs in response to FBS. Immunoblot analysis revealed that the expression of phosphorylated, that is, activated STAT3 (pSTAT3) was upregulated, after serum stimulation. (Figure 2a,b).

To make clear whether STAT3 activity is essential for the proliferation of NRCMs in the presence of FBS, we examined the effects of siRNA mediated STAT3 knockdown on NRCM proliferation (Figure 2c,d). Immunofluorescence microscopic analysis with anti‐Ki‐67 antibody revealed that STAT3 knockdown reduced the ratio of Ki‐67^+^ proliferating NRCMs (Figure 2e,f). Importantly, immunoblot analysis with anti‐Runx1 antibody showed that Runx1 expression was downregulated by STAT3 knockdown either in the presence or absence of FBS (Figure 2g,h). These results indicated that STAT3 activity is essential for the induction of Runx1 by FBS.

### 3.3 Runx1 is necessary and sufficient for NRCM proliferation in response to FBS

**FIGURE 3:**
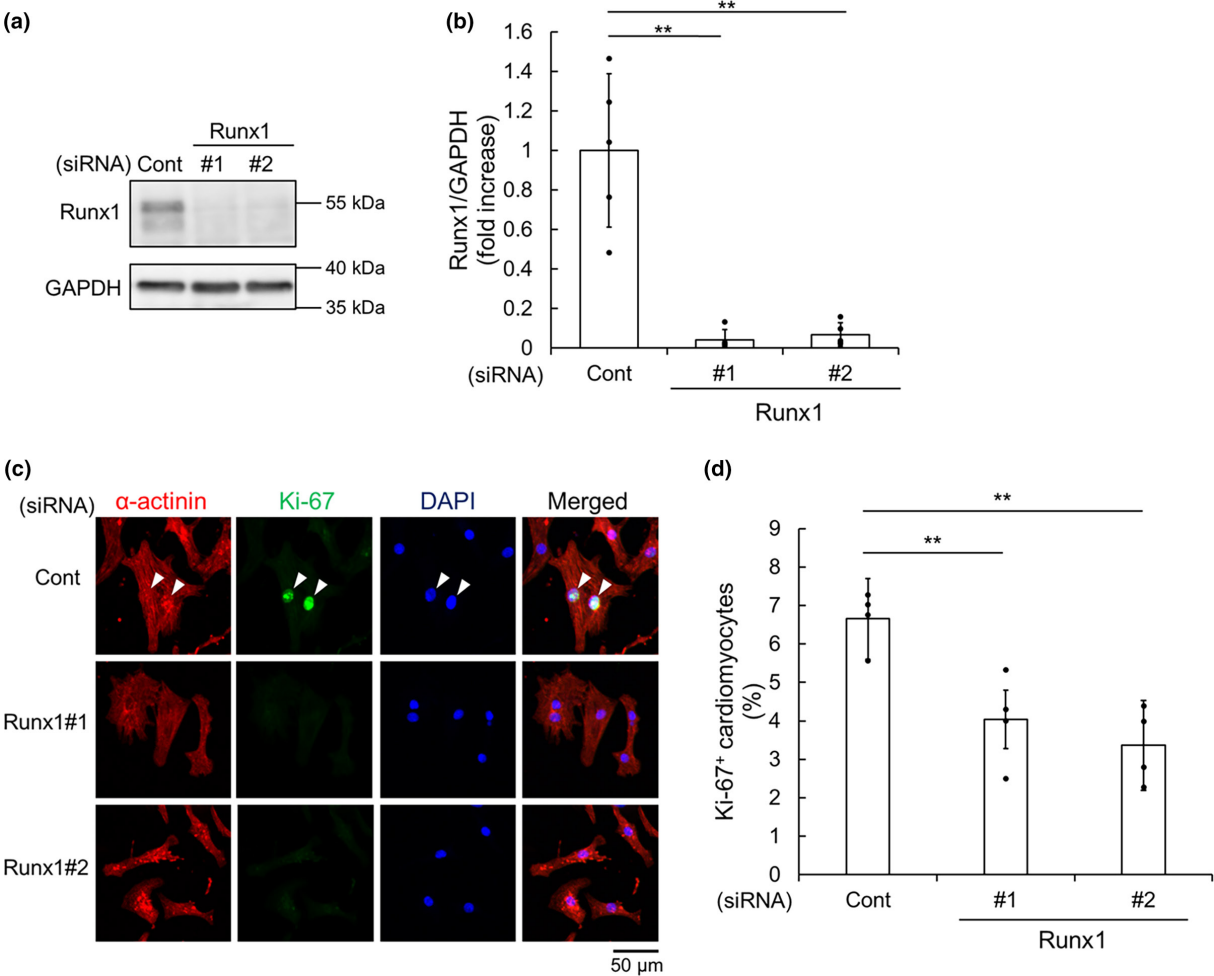
Runx1 is necessary for neonatal rat cardiomyocyte (NRCM) proliferation. (a–d) NRCMs were transfected with control siRNA (Cont) or siRNA for Runx1 (Runx1#1 and Runx1#2) at 10 nM. (a, b) Immunoblot analysis was performed with anti‐Runx1 and anti‐GAPDH antibodies. Representative images (a) and quantification of Runx1 (b) are shown. Data are shown as mean ± SD of fold increase. **p < 0.01 (*n* = 5) versus Cont by one‐way ANOVA followed by Dunnett test. (c, d) NRCMs were stained with anti‐sarcomeric α‐actinin and anti‐Ki‐67 antibodies. Nuclei were stained with DAPI. (c) Representative images are shown. Arrowheads indicate Ki‐67^+^ α‐actinin^+^ cells. (d) The percentage of Ki‐67^+^ α‐actinin^+^ cells per α‐actinin^+^ cells are shown. Data are shown as mean ± SD. **p < 0.01 (*n* = 4) versus Cont by one‐way ANOVA followed by Dunnett test.

To examine whether Runx1 is necessary for NRCM proliferation, we transfected control siRNA or siRNA targeting Runx1 to knockdown Runx1. Immunoblot analysis revealed that transfection with siRNA for Runx1 reduced the expression of Runx1 protein (Figure 3a,b). To determine the importance of Runx1 in the proliferation, NRCMs were transfected Runx1 siRNA and cultured in the presence of FBS. Immunofluorescence microscopic analysis revealed that the ratio of Ki‐67^+^ cardiomyocytes was significantly decreased (Figure 3c,d), indicating that Runx1 is essential for the proliferation of NRCMs.

**FIGURE 4:**
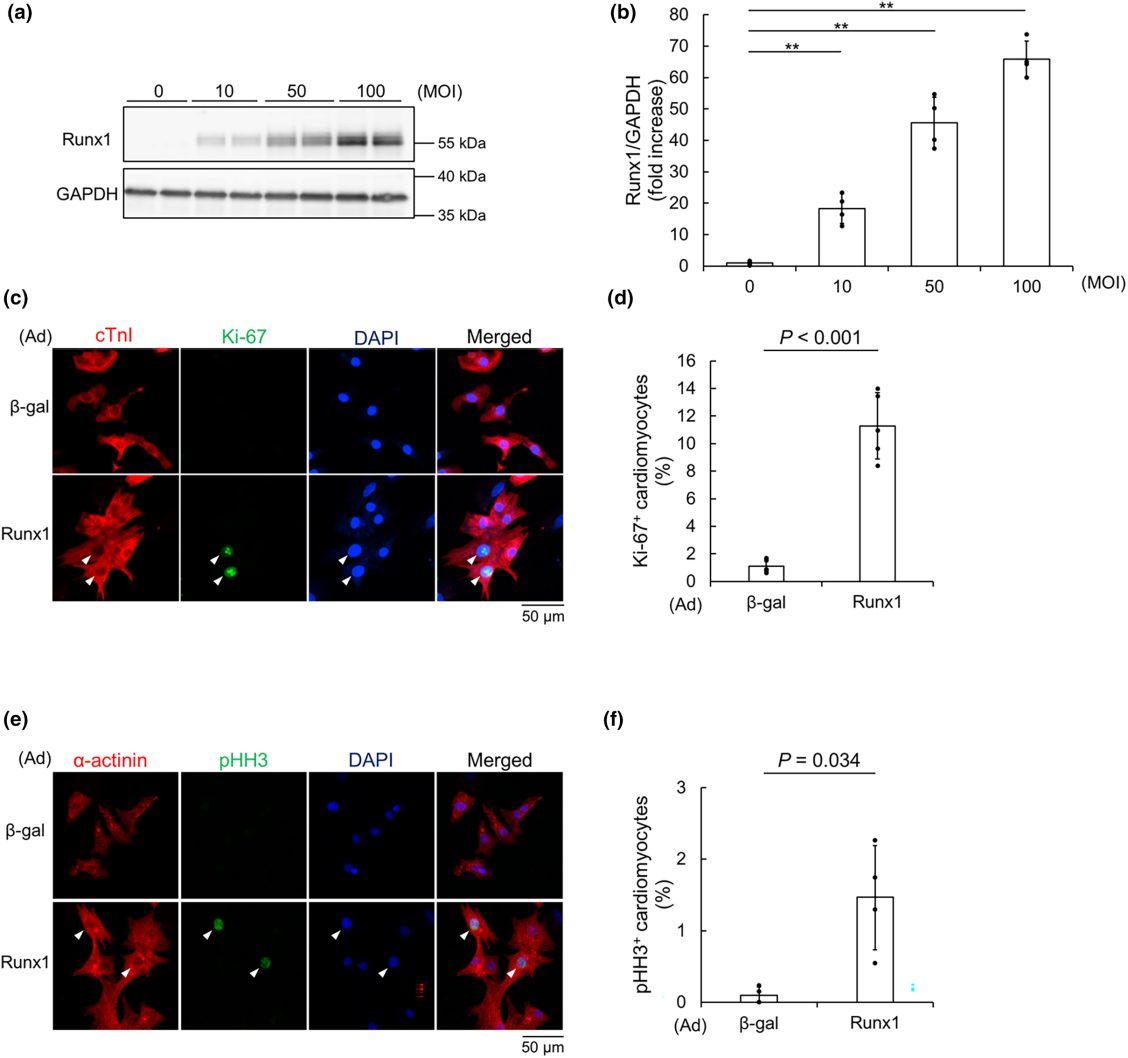
Runx1 overexpression induced neonatal rat cardiomyocyte (NRCM) proliferation. (a–d) NRCMs were transfected with adenovirus vector expressing Runx1 for the indicated concentration. (a, b) Immunoblot analysis was performed with anti‐Runx1 and anti‐GAPDH antibodies. Representative images (a) and quantification of Runx1 (b) are shown. Data are shown as mean ± SD of fold increase. **p < 0.01 (n = 4) versus 0 MOI by one‐way ANOVA followed by Dunnett test. (c–f) NRCMs were transfected with adenovirus vector expressing Runx1 or β‐galactosidase (β‐gal), as control at 100 MOI. (c, d) NRCMs were stained with anti‐cardiac Troponin I (cTnI) and anti‐Ki‐67 antibodies. Nuclei were stained with DAPI. (c) Representative images are shown. Arrowheads indicate Ki‐67^+^ cTnI^+^ cells. (d) The percentage of Ki‐67^+^ cTnI^+^ cells per cTnI^+^ cells are shown. Data are shown as mean ± SD. *p*‐value was calculated by unpaired, two‐tailed Welch's *t*‐test (*n* = 5). (e, f) NRCMs were stained with anti‐sarcomeric α‐actinin and anti‐phospho‐Histone H3 (pHH3) antibodies. Nuclei were stained with DAPI. (e) Representative images are shown. Arrowheads indicate pHH3^+^ α‐actinin^+^ cells. (f) The percentage of pHH3^+^ α‐actinin^+^ cells per α‐actinin^+^ cells are shown. Data are shown as mean ± SD. *p*‐value was calculated by unpaired, two‐tailed Welch's *t*‐test (*n* = 4).

To investigate whether the overexpression of Runx1 promotes NRCM proliferation, we used adenovirus vector expressing Runx1 (Ad‐Runx1). NRCMs were transfected with adenovirus vector at the various concentrations. Immunoblot analysis showed that adenoviral transfection increased Runx1 protein (Figure 4a,b). By using an adenovirus vector expressing β‐galactosidase (Ad‐β‐gal) as control, we evaluated the effects of Runx1 overexpression on NRCM proliferation. Immunofluorescence microscopic analysis revealed that the overexpression of Runx1 increased Ki‐67^+^ cardiomyocytes compared with Ad‐β‐gal (Figure 4c,d). In addition, the ratio of Ki‐67^+^ cardiomyocytes was significantly increased in a dose dependent manner (Figure S2A,B). Moreover, immunofluorescence microscopic analysis using anti‐phospho‐Histone H3 (pHH3) revealed that Runx1 overexpression increased pHH3^+^ cardiomyocytes (Figure 4e,f; Figure S2C,D), indicating Runx1 potentiates cardiomyocyte proliferation.

### 3.4 The expression of Runx1 induced the juvenilization of NRCMs

**FIGURE 5:**
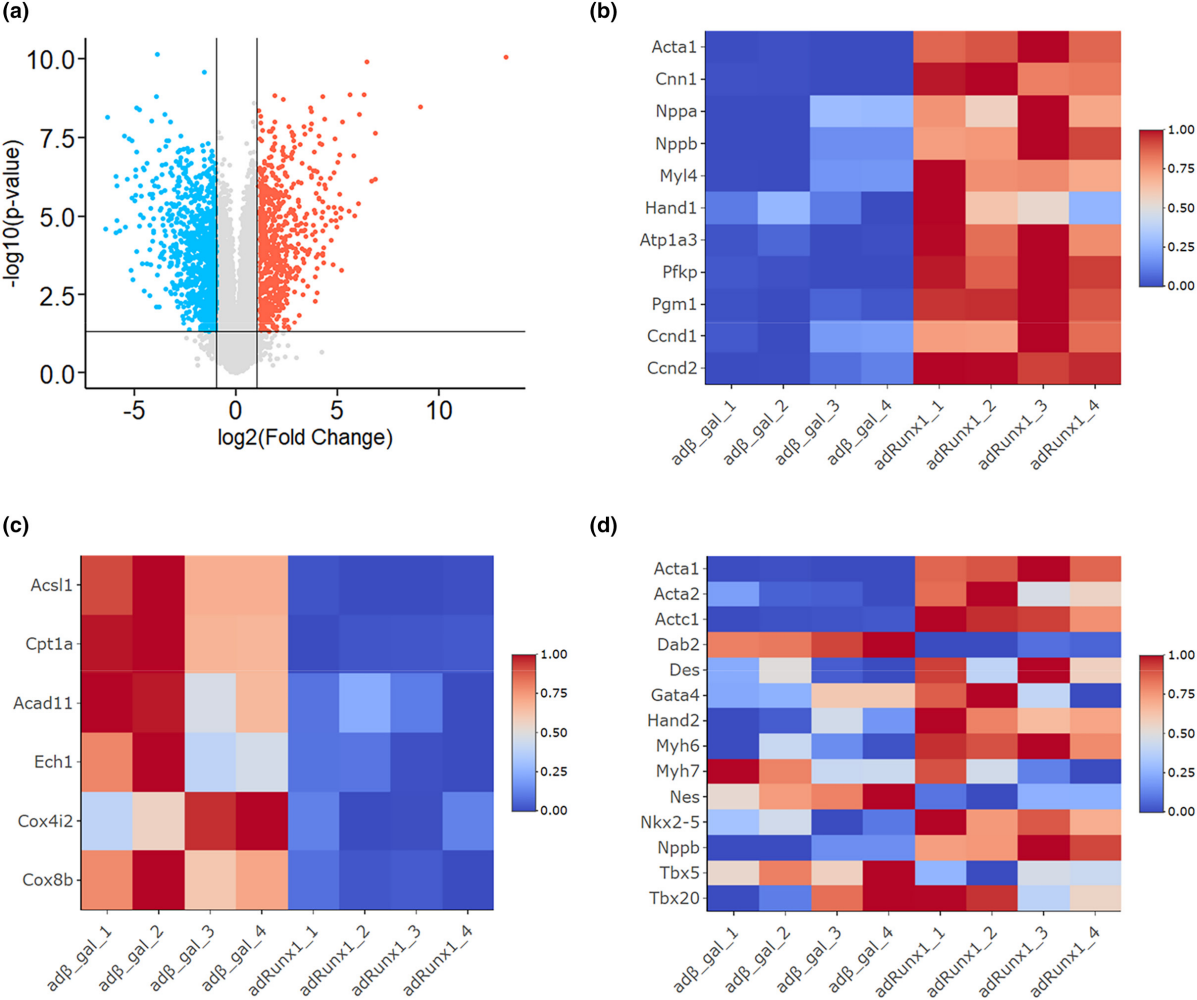
Overexpression of Runx1 juvenilized neonatal rat cardiomyocytes (NRCMs). (a–d) NRCMs were transfected with adenovirus vector expressing Runx1 or β‐gal, as control at 100 MOI. Total RNA was extracted, and RNA‐sequencing analysis was performed (*n* = 4 for each group). (a) Volcano plot shows differentially expressed genes. Red dots show the upregulated genes, and blue dots show the downregulated genes in Runx1 overexpressed NRCM (> 2‐fold change between Ad‐β‐gal and Ad‐Runx1, *p* < 0.05). (b) Heatmap shows the cardiac fetal genes that were upregulated by Runx1 overexpression. (c) Heatmap shows the genes associated with cardiac maturation genes that were downregulated by Runx1 overexpression. (d) Heatmap shows the expression of dedifferentiation marker genes.

To address the mechanisms of how Runx1 promoted NRCM proliferation, RNA‐sequencing analysis was performed to compare the gene expression profiles between NRCMs transfected with Ad‐Runx1 and those with Ad‐β‐gal. Genes with > 2‐fold change between Ad‐β‐gal and Ad‐Runx1 with a *p*‐value < 0.05 were identified as differentially expressed genes. The expression of 805 genes was upregulated, while that of 993 genes was downregulated by Runx1 overexpression (Figure 5a). Interestingly, cardiac fetal genes such as Acta1 (Chien et al., 1991), Cnn1 (Samaha et al., 1996), Nppa (Chien et al., 1991), Nppb (Barry et al., 2008), Myl4 (Hernandez et al., 2007), Hand1 (Okubo et al., 2021), Atp1a3 (Charlemagne et al., 1994), Pfkp (Shen et al., 2020), Pgm1 (Guo & Pu, 2020; Liu et al., 2023; Sim et al., 2015), Ccnd1 (Ikenishi et al., 2012), and Ccnd2 (Guo & Pu, 2020) were increased in Runx1‐overexpressing NRCMs (Figure 5b; Table S4). Conversely, genes associated with fatty acid oxidation or electron transport chain such as Acsl1 (Goldenberg et al., 2016), Cpt1a (Schlaepfer & Joshi, 2020), Acad11 (Swigonová et al., 2009), Ech1 (Huang et al., 2018), Cox4i2 (Mao et al., 2022), and Cox8b (DeLaughter et al., 2016) were decreased by Runx1 overexpression (Figure 5c; Table S5). Considering that fatty acid oxidation and electron transport chain are activated in mature cardiomyocytes, these data propose that the expression of Runx1 induced the juvenilization of NRCMs. Indeed, qRT‐RCR analysis revealed that some cardiac fetal genes were upregulated in NRCMs overexpressing Runx1 (Figure S3). Finally, we examined the effects of Runx1 on the expression of dedifferentiation markers (Zhu et al., 2021) (Figure 5d; Table S6). Interestingly, Acta1, Actc1, and Nppb are differentially increased by Runx1 overexpression. In addition, some markers significantly increased (*p*‐value < 0.05) in NRCMs transfected with Ad‐Runx1, though the change was less than 2‐fold.

## 4 DISCUSSION

**FIGURE 6:**
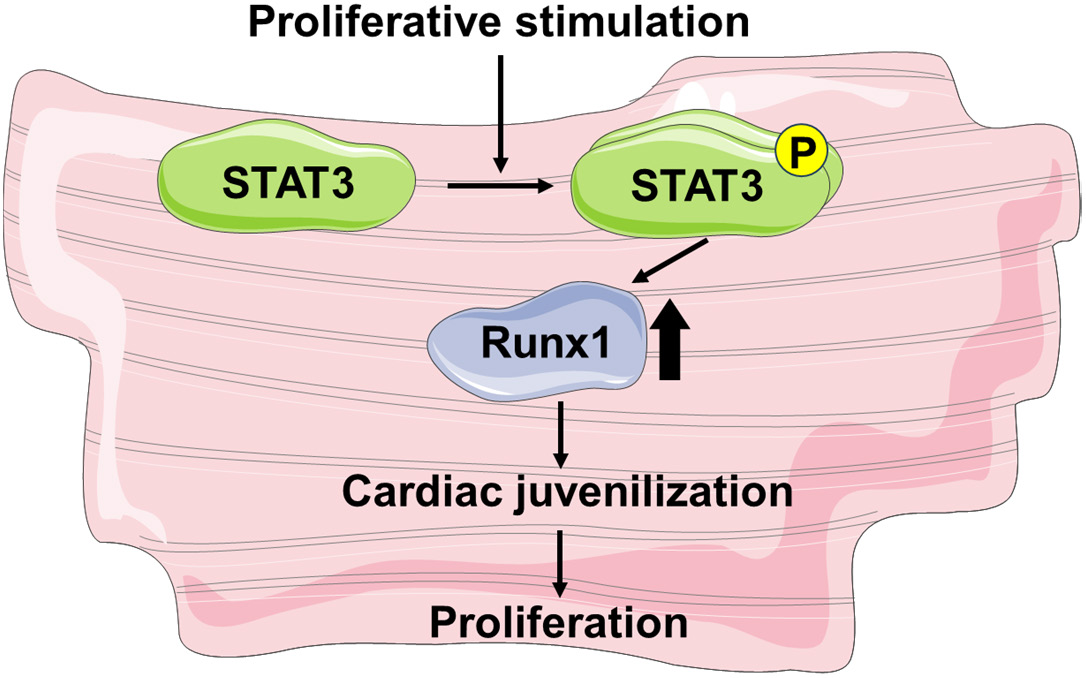
A working model of how signal transducer and activator of transcription 3 (STAT3)/Runx1 induces cardiomyocyte proliferation. In neonatal rat cardiomyocytes, STAT3 is activated in response to proliferating stimulation such as fetal bovine serum and upregulates Runx1 expression. Runx1 induces juvenilization and proliferation of cardiomyocyte. The figure was partly generated using Servier Medical Art, provided by Servier, licensed under a Creative Commons Attribution 3.0 unported license.

Cardiomyocytes are terminally differentiated cells. Prior to re‐entry into cell cycle, cardiomyocytes dedifferentiate into an immature phenotype, expressing dedifferentiation marker genes; however, the biological significance of these marker genes remains to be fully elucidated. Here, we demonstrated that the expression of Runx1, one of the dedifferentiation markers, was upregulated by FBS through STAT3. The induction of Runx1 was necessary and sufficient for cardiomyocyte proliferation. Importantly, the overexpression of Runx1 resulted in the upregulation of the genes characteristic of immature cardiomyocytes and in the downregulation of those of mature cardiomyocytes. These data indicated that STAT3‐mediated induction of Runx1 promotes cardiomyocyte proliferation by juvenilizing cardiomyocytes (Figure 6).

Activation of STAT3 in cardiomyocytes plays important roles in maintenance of cardiac homeostasis by promoting cell survival and angiogenesis (Obana et al., 2010). Interestingly, recent studies have shown the importance of STAT3 in cardiomyocyte proliferation. In zebrafish, STAT3 activation is essential for cardiomyocyte proliferation in heart dissection model (Fang et al., 2013). We also revealed that STAT3 is activated in adult mammalian cardiomyocytes in myocarditis and contributes to cardiomyocyte proliferation and, as a result, to the functional and structural recovery from myocardial injury (Miyawaki et al., 2017). In this study, we showed that STAT3 mediates the induction of Runx1 by FBS. However, it remains to be addressed how activated STAT3 induced Runx1 in cardiomyocytes. A recent study identified the STAT3 binding site in the promotor region of Runx1. Importantly, STAT3 induced the promotor activity of the *Runx1* gene by binding to its promotor region in hepatocytes both in vitro and in vivo (Zhang et al., 2023), demonstrating that the expression of Runx1 is directly regulated by STAT3. The data presented here propose that Runx1 is a downstream target of STAT3 in NRCMs and that STAT3/Runx1 pathway is, at least partially, responsible for cardiomyocyte proliferation.

Runx1 has been identified as a key transcriptional regulator of hematopoiesis (Lam & Zhang, 2012). Since the alteration of *Runx1* gene results in the leukemia, it is widely accepted that Runx1 is involved in cell proliferation. Recently, it was reported that Runx1 overexpression increased mononuclear diploid cardiomyocytes (MNDCMs) and promoted cardiomyocyte proliferation in adult murine hearts (Swift et al., 2023). Consistent with their hypothesis, the expression of TNNI3 interacting kinase (TNNI3K), whose inhibition increases NMDCMs, was significantly suppressed in cardiomyocytes transfected with Ad‐Runx1 (−1.560 fold, *p* = 7.99E‐4), according to our RNA‐sequencing data. However, we cannot evaluate the importance of cardiomyocyte ploidy in NRCM in our experimental condition because we used neonatal cardiomyocyte culture that mainly consists of MNDCMs. It is possible that Runx1 increases the proliferative activity of NRCMs without modulating cardiomyocyte ploidy.

Originally, cardiomyocyte dedifferentiation was observed before proliferation in the heart dissection model of zebrafish (Jopling et al., 2010). Similar to zebrafish, recent studies demonstrated that mammalian cardiomyocytes also undergo dedifferentiation prior to proliferation (Wang et al., 2017). Dedifferentiation of cardiomyocytes is defined as sarcomere disassembly, metabolic switch from fatty acid oxidation to glycolysis and juvenile‐like cytoskeleton gene expression (Zhu et al., 2021). From the viewpoint of this definition, it is important that Runx1 overexpression upregulated the expression of cardiac fetal genes and downregulated that of fatty acid oxidation related genes. Moreover, the overexpression of Runx1 significantly increased several dedifferentiation markers. Based on these findings, it could be proposed that Runx1 is not only a biological marker but also a key molecule responsible for the dedifferentiation of cardiomyocytes, though the transcription factors that interact with Runx1 should be identified in order to make clear the molecular mechanism more in detail.

Cardiomyocytes largely lose the proliferative activity, though the functional significance of Runx1 in cell cycle exit is not fully addressed. Interestingly, epigenetic gene modifications such as DNA methylation patterns change from neonate to adult (Guo & Pu, 2020). In particular, DNA methylation of Runx1 target genes increases (Górnikiewicz et al., 2016), indicating that Runx1 signaling pathway is silenced. Considering that adult cardiomyocytes proliferate in myocarditis in a STAT3‐dependent manner (Miyawaki et al., 2017), it would be informative to examine whether STAT3/Runx1 is activated by inflammatory reaction in adult cardiomyocytes.

In conclusion, we demonstrated that STAT3‐mediated induction of Runx1 promotes cardiomyocyte proliferation by juvenilizing cardiomyocytes. Our data presented here provide a novel insight into the molecular mechanisms of cardiomyocyte dedifferentiation and proliferation.

## AUTHOR CONTRIBUTIONS

Shota Suzuki, Shota Tanaka, Masanori Obana, and Yasushi Fujio conceived and designed research; Shota Suzuki, Yusuke Kametani, Ayaka Umeda, Kosuke Nishinaka, and Kaho Egawa performed experiments; Shota Suzuki analyzed data; Shota Suzuki, Shota Tanaka, Yoshiaki Okada, Masanori Obana, and Yasushi Fujio interpreted results of experiments; Shota Suzuki prepared figures; Shota Suzuki drafted manuscript; Shota Suzuki, Shota Tanaka, Yoshiaki Okada, Masanori Obana, and Yasushi Fujio edited and revised manuscript; Shota Suzuki, Shota Tanaka, Yusuke Kametani, Ayaka Umeda, Kosuke Nishinaka, Kaho Egawa, Yoshiaki Okada, Masanori Obana, and Yasushi Fujio approved final version of manuscript.

## FUNDING INFORMATION

This study was partially supported by MEXT/JSPS KAKENHI Grant 22K15277 to S.T., and Grant‐in‐Aid for Japan Society for the Promotion of Science Research Fellowships for Young Scientists to Y.K. (Grant number 23KJ1461). This study was also partially supported by Platform Project for Supporting Drug Discovery and Life Science Research (Basis for Supporting Innovative Drug Discovery and Life Science Research (BINDS)) from AMED under Grant Number JP23ama121052 and JP23ama121054 and by Hyogo Science and Technology Association.

## CONFLICT OF INTEREST STATEMENT

The authors have no conflict of interest to disclose.

## EHICS STATEMENT

Protocols of animal study were approved by the ethics of the Committee of Osaka University and Institutional Animal Care (approval number: Douyaku, R03‐16‐5). Genetic modification experiments were conducted in accordance with the regulations for genetic modification experiments at Osaka University (approval number: (I)04809).

## Supporting information


Figure S1.

Table S1.

## Data Availability

The data that support the findings of this study are available from the corresponding authors, Y. F., upon reasonable request.
