# Supplementary material for: Runx1 is upregulated by STAT3 and promotes proliferation of neonatal rat cardiomyocytes
Source: Physiol Rep. 2023 Dec 1;11(23):e15872. doi: 10.14814/phy2.15872 (PMC10691971; doi:10.14814/phy2.15872)
Supplement: Supplementary file 1 — Figure S1. [file PHY2-11-e15872-s002.pdf]

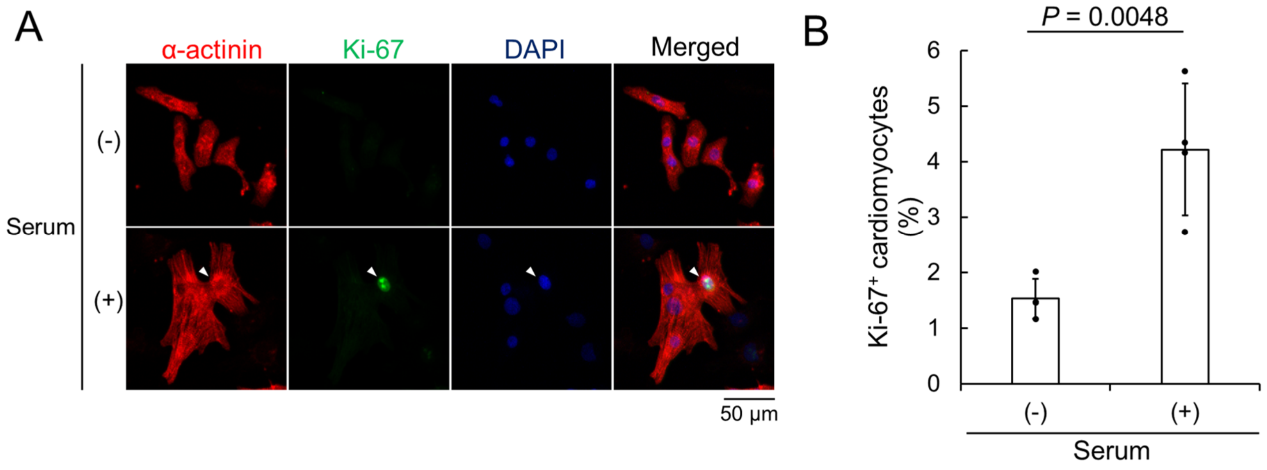

Supplemental Figure S1. FBS promoted NRCM proliferation.

(A, B) NRCMs were cultured in the presence or absence of FBS and stained with anti-sarcomeric α-actinin and anti-Ki-67 antibodies. Nuclei were stained with DAPI. (A) Representative images are shown. Arrowheads indicate Ki-67^{+} α-actinin^{+} cells. (B) The percentage of Ki-67^{+} α-actinin^{+} cells per α-actinin^{+} cells are shown. Data are shown as mean±SD. *P*-value was calculated by unpaired, two-tailed Student's *t*-test.

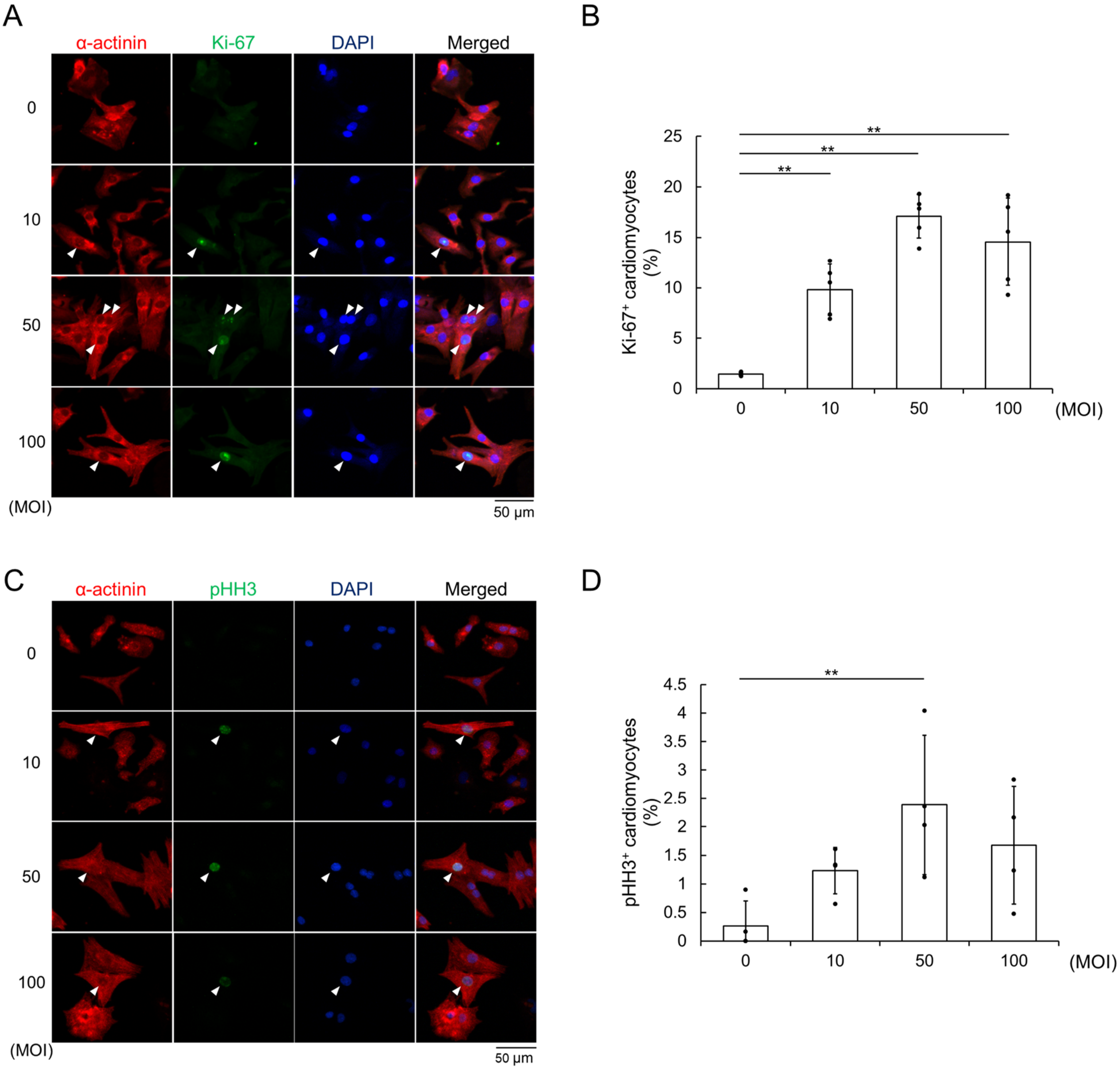

Supplemental Figure S2. Runx1 overexpression induced NRCM proliferation.

(A-D) NRCMs were transfected with adenovirus vector expressing Runx1 for the indicated concentration. (A, B) NRCMs were stained with anti-sarcomeric α-actinin and anti-Ki-67 antibodies. Nuclei were stained with DAPI. (A) Representative images are shown. Arrowheads indicate Ki-67^{+} α-actinin^{+} cells. (B) The percentage of Ki-67^{+} α-actinin^{+} cells per α-actinin^{+} cells are shown. Data are shown as mean±SD. ***P* < 0.01 (n = 5) vs. 0 MOI by one-way ANOVA followed by Dunnett test. (C, D) NRCMs were stained with anti-sarcomeric α-actinin and anti-pHH3 antibodies. Nuclei were stained with DAPI. (C) Representative images are shown. Arrowheads indicate pHH3^{+} α-actinin^{+} cells. (D) The percentage of pHH3^{+} α-actinin+ cells per α-actinin^{+} cells are shown. Data are shown as mean±SD. **P* < 0.05 (n = 4) vs. 0 MOI by one-way ANOVA followed by Dunnett test.

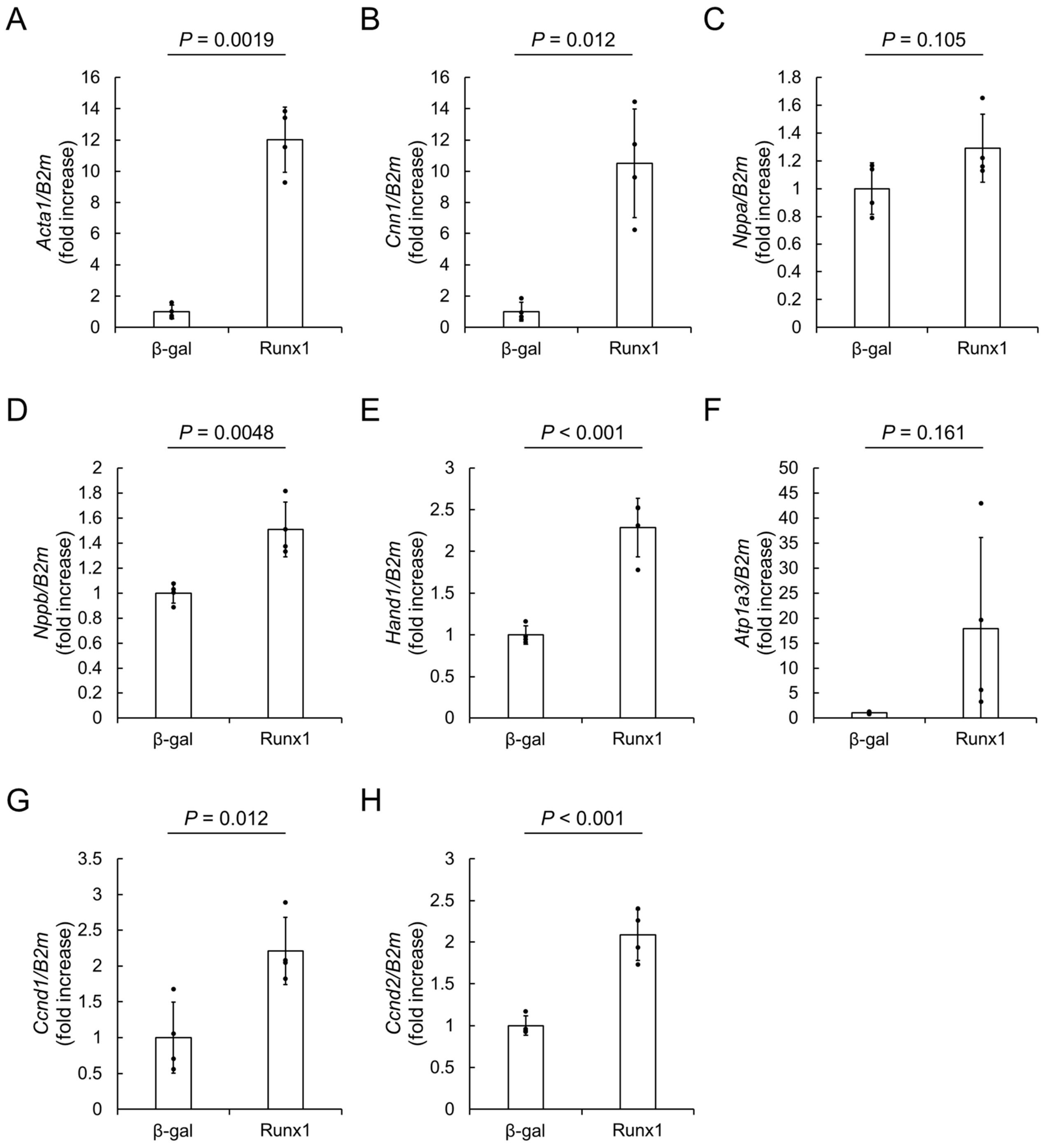

Supplemental Figure S3. Runx1 overexpression upregulated cardiac fetal gene expression.

(A-H) NRCMs were transfected with adenovirus vector expressing Runx1 or β-gal, as control at 100 MOI. The expression of cardiac fetal gene mRNA was quantified by qRT-PCR and normalized to that of *B2m*. Data are shown as mean±SD of fold change. (A, B, F) *P*-value was calculated by unpaired, two-tailed Welch's *t*-test. (C, D, E, G, H) *P*-value was calculated by unpaired, two-tailed Student's *t*-test.
