# Supplementary material for: Runx1 is upregulated by STAT3 and promotes proliferation of neonatal rat cardiomyocytes
Source: Physiol Rep. 2023 Dec 1;11(23):e15872. doi: 10.14814/phy2.15872 (PMC10691971; doi:10.14814/phy2.15872)
Supplement: Supplementary file 2 — Table S1. [file PHY2-11-e15872-s001.docx]

**Supplemental Table S1. Antibodies used for immunoblot analyses in the present study.**

| **Target** | **Manufacturer** | **Cat.#** | **Dilution** |
| --- | --- | --- | --- |
| Runx1 | Santa Cruz | sc-365644 | 1/500 |
| GAPDH | Miliipore | MAB374 | 1/1000 |
| STAT3 | Cell Signaling | 4904 | 1/1000 |
| pSTAT3 (Y705) | Cell Signaling | 9131 | 1/1000 |
| HRP-linked  anti-mouse IgG | Jackson ImmunoReserch | 115-035-062 | 1/1000 |
| HRP-linked anti-rabbit IgG | cell signaling | 7074 | 1/1000 |

**Supplemental Table S2. siRNA used in the present study.**

| **Name** | **Manufacturer** | **Cat. #** | **Direction** | **Sequence** |  |
| --- | --- | --- | --- | --- | --- |
| Cont | Merck | SIC001-10NMOL | Merck's proprietary sequence | |  |
|  |  |  |  |  |  |
| Runx1#1 | Merck | SASI_Rn01_00092605 | forward | 5'-CAAGUUGCCACCUACCAUA-3' |  |
|  |  |  | reverse | 5'-UAUGGUAGGUGGCAACUUG-3' |  |
| Runx1#2 | Merck | SASI_Rn02_00261774 | forward | 5'-CCUACCAUAGAGCCAUCAA-3' |  |
|  |  |  | reverse | 5'-UUGAUGGCUCUAUGGUAGG-3' |  |
| Stat3 | invitrogen | 10620312 | forward | 5'-GACUGGUGUCCAGUUUACCACGAAA-3' |  |
|  |  |  | reverse | 5'-UUUCGUGGUAAACUGGACACCAGUC-3' |  |

**Supplemental Table S3. Antibodies used for immunofluorescence microscopic analyses in the present study.**

| **Target** | **Manufacturer** | **Cat.#** | **Dilution** |
| --- | --- | --- | --- |
| α-actinin | Sigma-Aldrich | A7811-2ML | 1/1000 |
| Ki-67 | invitrogen | 14-5698-82 | 1/600 |
| DAPI | Nacalai Tesque | 1134-56 | 1/400 |
| cTnI | abcam | ab56357 | 1/1000 |
| pHH3 (S10) | Cell Signaling | 9701 | 1/200 |
| Alexa Fluor 488 conjugated  goat anti rat | abcam | ab150157 | 1/400 |
| Alexa Fluor 488 conjugated  goat anti rabbit | invitrogen | A11034 | 1/400 |
| Alexa Fluor 488 conjugated  donkey anti rat | invitrogen | A21208 | 1/400 |
| Alexa Fluor 546 conjugated  goat anti mouse | invitrogen | A11030 | 1/400 |
| Alexa Fluor 546 conjugated  goat anti mouse | invitrogen | A11056 | 1/400 |

**Supplemental Table S4. The fold change, *P*-value, and *q*-value of differently expressed cardiac fetal genes.**

| **Gene name** | **Fold Change** | ***P*-value** | ***q*-value** |
| --- | --- | --- | --- |
| Acta1 | 19.183 | 1.64E-16 | 6.18E-13 |
| Cnn1 | 16.506 | 8.80E-15 | 1.10E-11 |
| Nppa | 3.663 | 1.41E-04 | 0.0005362 |
| Nppb | 3.590 | 8.54E-08 | 1.11E-06 |
| Myl4 | 3.620 | 4.82E-07 | 4.64E-06 |
| Hand1 | 2.156 | 9.73E-04 | 0.0027668 |
| Atp1a3 | 6.509 | 9.40E-12 | 1.16E-09 |
| Pfkp | 2.302 | 3.48E-11 | 3.22E-09 |
| Pgm1 | 2.435 | 2.28E-11 | 2.41E-09 |
| Ccnd1 | 2.126 | 1.84E-07 | 2.08E-06 |
| Ccnd2 | 2.716 | 2.56E-10 | 1.32E-08 |

**Supplemental Table S5. The fold change, *P*-value, and *q*-value of differently expressed genes associated with fatty acid oxidation or electron transport system.**

| **Gene name** | **Fold Change** | ***P*-value** | ***q*-value** |
| --- | --- | --- | --- |
| Acsl1 | -2.566 | 5.17E-10 | 2.20E-08 |
| Cpt1a | -2.346 | 3.78E-09 | 9.78E-08 |
| Acad11 | -2.386 | 9.61E-06 | 5.58E-05 |
| Ech1 | -3.394 | 1.44E-06 | 1.13E-05 |
| Cox4i2 | -2.625 | 9.15E-05 | 0.0003706 |
| Cox8b | -2.361 | 5.20E-09 | 1.25E-07 |

**Supplemental Table S6. The fold change, *P*-value, and *q*-value of differently expressed dedifferentiation marker genes.**

| **Gene name** | **Fold Change** | ***P*-value** | ***q*-value** |
| --- | --- | --- | --- |
| Acta1 | 19.183 | 1.64E-16 | 6.18E-13 |
| Acta2 | 1.332 | 1.24E-04 | 0.0004803 |
| Actc1 | 3.299 | 1.11E-11 | 1.28E-09 |
| Dab2 | -1.571 | 5.77E-08 | 8.22E-07 |
| Des | 1.213 | 6.00E-03 | 0.0133443 |
| Gata4 | 1.016 | 7.08E-01 | 0.769126 |
| Hand2 | 1.271 | 5.33E-04 | 0.0016617 |
| Myh6 | 1.468 | 5.10E-05 | 0.0002279 |
| Myh7 | -1.090 | 3.20E-01 | 0.4016108 |
| Nes | -1.477 | 4.14E-05 | 0.0001915 |
| Nkx2-5 | 1.277 | 4.00E-04 | 0.0013036 |
| Nppb | 3.590 | 8.54E-08 | 1.11E-06 |
| Tbx5 | -1.202 | 1.33E-02 | 0.026625 |
| Tbx20 | 1.070 | 2.74E-01 | 0.3526765 |
